# Transcription Landscape of the Early Developmental Biology in Pigs

**DOI:** 10.3390/ani11051443

**Published:** 2021-05-18

**Authors:** Susana A. Teixeira, Daniele B. D. Marques, Thaís C. Costa, Haniel C. Oliveira, Karine A. Costa, Eula R. Carrara, Walmir da Silva, José D. Guimarães, Mariana M. Neves, Adriana M. G. Ibelli, Maurício E. Cantão, Mônica C. Ledur, Jane O. Peixoto, Simone E. F. Guimarães

**Affiliations:** 1 Department of Animal Science, Universidade Federal de Viçosa, Viçosa 36570-000, MG, Brazil; susana.teixeira@ufms.br (S.A.T.); danielebdiniz@gmail.com (D.B.D.M.); thaisccosta12@gmail.com (T.C.C.); hanielcedraz@gmail.com (H.C.O.); kryneacosta@yahoo.com.br (K.A.C.); eula.carrara@ufv.br (E.R.C.); walmir@ufv.br (W.d.S.); 2 Department of Veterinary Medicine, Universidade Federal de Viçosa, Viçosa 36570-000, MG, Brazil; jdguima@ufv.br; 3 Department of General Biology, Universidade Federal de Viçosa, Viçosa 36570-000, MG, Brazil; mariana.mneves@ufv.br; 4 Embrapa Suínos e Aves, Concordia 89715-899, SC, Brazil; adriana.ibelli@embrapa.br (A.M.G.I.); mauricio.cantao@embrapa.br (M.E.C.); monica.ledur@embrapa.br (M.C.L.); jane.peixoto@embrapa.br (J.O.P.)

**Keywords:** organogenesis, swine, prenatal development, RNA-seq

## Abstract

**Simple Summary:**

A complete and exploratory landscape of developmental biology during mammalian organogenesis is needed to improve livestock production and contribute with comparative developmental studies in other relative species, including humans. Since transcriptome analysis is suitable to understand systemic context and genomic interactions, we evaluated the main transcriptional changes during overall organogenesis comparing fetal and embryonic stages through RNA-seq analysis, using the pig as model. The differentially expressed genes between pig fetuses and embryos highlight the main biological events and pathways required for primary organs and body structure development at the embryonic stage, the biological events and pathways related to growth and specialization of these organs at the fetal stage, as well as the molecular mechanisms involved in embryonic to fetal transition. This exploratory transcriptional landscape contributes to improving our knowledge of the crucial biological events during normal prenatal development, which is relevant in prenatal programing studies as well as in livestock production.

**Abstract:**

Since pre- and postnatal development are programmed during early prenatal life, studies addressing the complete transcriptional landscape during organogenesis are needed. Therefore, we aimed to disentangle differentially expressed (DE) genes between fetuses (at 35 days old) and embryos (at 25 days old) through RNA-sequencing analysis using the pig as model. In total, 1705 genes were DE, including the top DE *IBSP*, *COL6A6*, *HBE1*, *HBZ*, *HBB*, and *NEUROD6* genes, which are associated with developmental transition from embryos to fetuses, such as ossification, skeletal muscle development, extracellular matrix organization, cardiovascular system, erythrocyte differentiation, and neuronal system. In pathway analysis, embryonic development highlighted those mainly related to morphogenic signaling and cell interactions, which are crucial for transcriptional control during the establishment of the main organs in early prenatal development, while pathways related to myogenesis, neuronal development, and cardiac and striated muscle contraction were enriched for fetal development, according to the greater complexity of organs and body structures at this developmental stage. Our findings provide an exploratory and informative transcriptional landscape of pig organogenesis, which might contribute to further studies addressing specific developmental events in pigs and in other mammals.

## 1. Introduction

Early prenatal development is highly susceptible to developmental abnormalities, which may lead to impaired survival of conceptuses [1]. In mammals, the prenatal development is characterized by three main stages. First, the pre-implantation period that comprises from fertilization to attachment of the embryo into the uterine wall. Second, the organogenesis period in which the main tissues, organs, and body structures are formed. Finally, the third stage is marked by the refinement and growth of these organs, structures and tissues, characterizing the fetal period [1,2,3]. During embryogenesis, the development of complex and multicellular tissues and organs requires a well-orchestrated set of cellular events, involving cell–cell signaling and an intrinsic transcriptional program [4].

In pigs, a substantial increase in prenatal losses during the post-implantation period has been reported as a consequence of uterine crowding, mainly in highly prolific breeds [5,6]. In contrast, the surviving conceptuses under uterine crowding conditions may present intrauterine growth restriction (IUGR) [6], resulting in developmental biology impairments, such as a reduced number of pancreatic islets, kidney glomeruli, and muscle fibers, which, ultimately, compromise the productivity and functionality of the affected progeny in postnatal life [7]. As many of these adverse effects on postnatal life are usually programmed in the prenatal development [6], the knowledge of the key genes and their signaling pathways that regulate biological events related to organogenesis may provide a valuable support for studies addressing the causes and consequences of prenatal programming in the context of molecular biology, as well as the improvement of swine and other livestock production.

Several studies have reported on transcriptional changes during prenatal development in pigs. However, most of them have been conducted in embryos or endometrium in the pre-implantation period [8,9], whereas others have addressed the post implantation events mainly related to myogenesis from a limited set of genes obtained from microarray experiments [10,11]. Therefore, the complete background regarding the main transcriptional changes and their association with the biological events during pig organogenesis remain poorly understood.

Based on this, a high-throughput transcriptome analysis was used to identify differentially expressed (DE) genes between early fetuses (35 days old) and embryos (25 days-old), to obtain an exploratory and complete transcriptional landscape during these development stages. In addition, we aimed to disentangle some of the biological processes and pathways associated with developmental biology, which can contribute to further studies addressing a specific biological event in pigs and also in other mammals.

## 2. Materials and Methods

### 2.1. Animal Experiment

The experimental protocol has followed ethical principles in animal research (CONCEA, 2016) and was approved by the Ethical Committee on Animal Use of Universidade Federal de Viçosa (UFV), Minas Gerais, Brazil (protocol no. 06/2017).

The present study is part of a whole experiment initially designed to understand the effects of maternal dietary L-arginine supplementation on pig embryos at 25 days old and fetuses at 35 days old [12,13], as well as compare organogenesis. Particularly, in the current study, we describe the transcriptional changes that occur during the development stages instead of the effects of L-arginine supplementation. Therefore, only data from embryos and fetuses from non-supplemented gilts (*n* = 3 embryos from each three non-supplemented gilts at 25 days of gestation and *n* = 3 fetuses from each three non-supplemented gilts at 35 days of gestation), totalizing 9 embryos and 9 fetuses, were used for the analyses. After sampling, each embryo and fetus was entirely and separately macerated in liquid nitrogen for RNA-sequencing procedures. Additional details regarding the experimental design, animal management, and sample collection and preparation for RNA-sequencing (RNA-seq) analyses are available in Supplementary Materials S1.

### 2.2. RNA Extraction, Library Preparation and Sequencing

The procedures of RNA extraction, library preparation, and sequencing were performed as previously described in [14]. Briefly, 100 mg of whole macerated conceptuses were mixed to 1 mL Trizol^®^ reagent (Invitrogen, San Diego, CA, USA) and the Qiagen RNeasy^®^ Mini kit (Qiagen, Hilden, Germany) was used for total RNA isolation, following the manufacturer’s instructions. Afterwards, the QUBIT fluorometer (Thermo Scientific, Waltham, MA, USA) was used to quantify the extracted RNA and its integrity was determined in 1.0% agarose gel. In addition, the RNA integrity number (RIN) was evaluated using an Agilent 2100 BioAnalyzer (Agilent Technologies, Santa Clara, CA, USA) and samples with RIN > 8 were kept for further analyses. The cDNA libraries of 35 conceptuses samples were prepared using the truSeq Stranded mRNA Library Prep Kit (Illumina, Inc., San Diego, CA, USA), followed by purification of the poly-A tail using 2 µg of total RNA starting material, according to the manufacturer’s recommendations. Sequencing was performed in an Illumina HiSeq2500 (Illumina, Inc.; San Diego CA, EUA), following the 2 × 100 bp paired-end protocol, at the Functional Genomics Center, ESALQ, Universidade de São Paulo, Piracicaba, SP, Brazil.

From the 35 sequenced samples, 18 conceptuses samples from the non-supplemented gilts (*n* = 9 embryos at 25 days old and *n* = 9 fetuses at 35 days old) were used in the present study to obtain an exploratory transcriptional landscape occurring during the development of pig embryos and fetuses.

### 2.3. RNA-Sequencing Data Analysis

The sequence data were deposited in the Sequence Read Archive of the NCBI databases under Bioproject number PRJNA576701 (https://www.ncbi.nlm.nih.gov/sra/?term=PRJNA576701, accessed on 10 October 2019) and the 18 conceptuses samples used in the present study are under Biosample numbers SAMN13003040, SAMN13003041, SAMN13003042, SAMN13003043, SAMN13003044, SAMN13003045, SAMN13003046, SAMN13003047, SAMN13003048, SAMN13003049, SAMN13003050, SAMN13003051, SAMN13003052, SAMN13003053, SAMN13003054, SAMN13003055, SAMN13003056 and SAMN13003057.

Raw reads (FASTQ) were trimmed with Trimmomatic tool [15], version 0.38, using the Phred quality score ≥20 and reads length ≥70 bp as parameters. Then, the remaining sequences were mapped against the pig reference genome (*Sus scrofa*, v. 11.1) using Hisat2 software [16], version 2.1.0, and the read counting was performed with HTSeq count software [17], version 0.11.2, using Ensembl annotation (release 95). All these analyses were performed following the BAQCOM pipeline [18]. The conceptuses’ sexes were determined according to Teixeira et al. [14].

The DE genes between pig fetuses and embryos were accessed using the limma [19] package in the R environment [20], with the sex effect used as an adjustment factor in this analysis. Since the DE genes were obtained through contrasts between fetal and embryonic developmental stages, the positive and negative log2-fold changes (logFC) indicate genes upregulated in fetuses and embryos, respectively. Genes were considered DE using a set of significance thresholds, such as an adjusted *p*-value for the false discovery rate (FDR) < 0.05, according to the Benjamini–Hochberg method [21], and |logFC| > 0.5.

### 2.4. Functional Analyses

The functional annotation of DE genes was performed in the Database for Annotation, Visualization, and Integrated Discovery (DAVID) tool, version 6.8 (https://david.ncifcrf.gov/tools.jsp, accessed on 12 January 2020) [22], using all DE genes as input list and the *Sus scrofa* genome (version 11.1) as the background list. The biological processes (BP) categories were considered as functionally enriched at FDR < 0.05 after the Benjamini–Hochberg procedure [21] and submitted to REVIGO [23] to summarize and visualize the gene ontology (GO) terms. The Toppcluster [24] online tool (http://toppcluster.cchmc.org, accessed on 20 January 2020) was used to visualize the enriched pathways (FDR < 0.05) related to the set of annotated genes upregulated in embryos (Cluster 1) and fetuses (Cluster 2). Finally, the visualization of the functional enrichment from the GO terms and network pathways was improved using Cytoscape [25], version 3.8.0.

## 3. Results

### 3.1. RNA-Sequencing Data

From the whole transcriptome of conceptuses samples (*n* = 9 embryos and *n* = 9 fetuses), an average of 29.4 million reads/samples was generated. After quality control, an average of 26.1 million reads/samples was kept. Moreover, an average of 98.7% of the reads were mapped against the pig reference genome (*Sus scrofa*, v.11.1, Ensembl 95).

**Figure 1:**
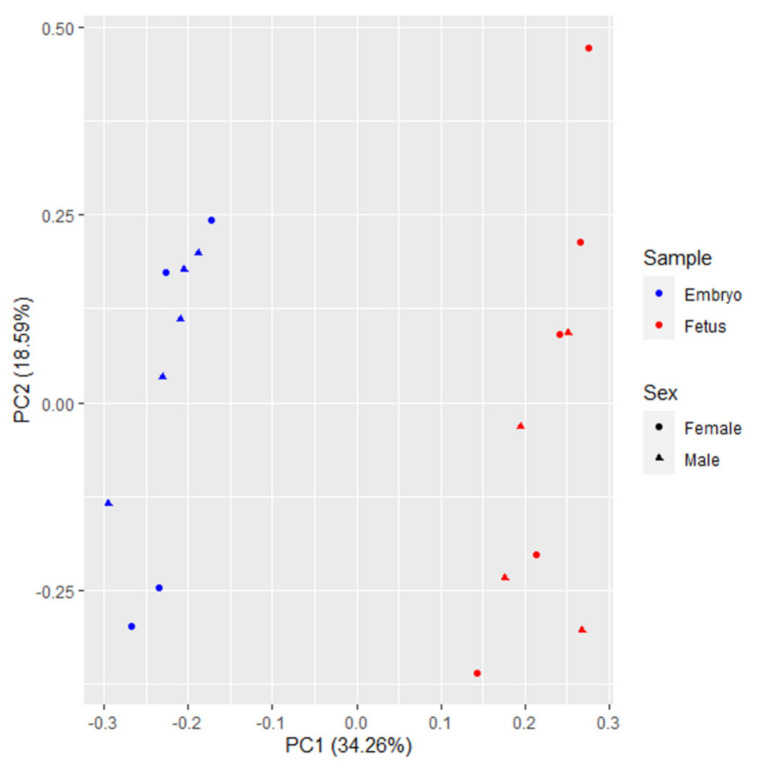
Principal component analysis of embryo samples at 25 days old (*n* = 9) and fetuses’ samples at 35 days old (*n* = 9), based on all genes detected in the pig’s prenatal transcriptome. Conceptuses from different prenatal ages show an evident separation.

A principal component analysis for all genes expressed in conceptuses samples (14,587 in total) was performed and revealed that conceptuses of the same developmental stage were grouped together, and high distances were observed between embryonic and fetal stages (Figure 1). This result reflects the accentuated transcriptional differences between the prenatal ages evaluated in the current study.

### 3.2. Differentially Expressed Genes between Fetuses and Embryos

**Figure 2:**
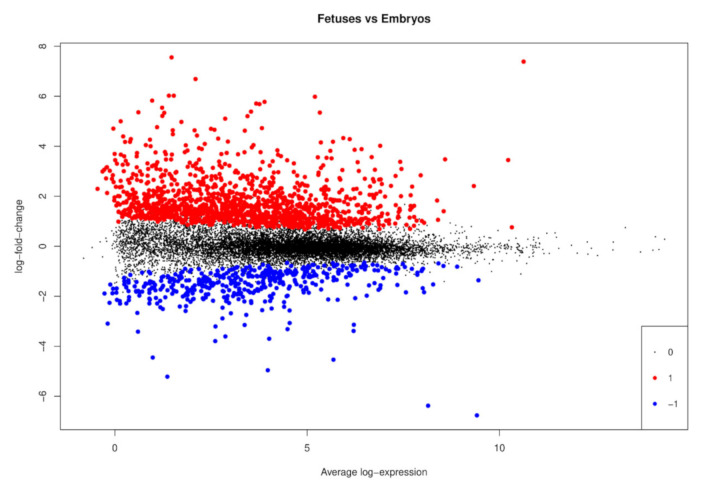
Mean-difference plot showing the log2-fold change (logFC) and average abundance of each differentially expressed (DE) gene between pig fetuses and embryos. The downregulated genes in pig fetuses were described as upregulated in pig embryos. Significantly (adjusted *p*-value < 0.05 from Benjamini–Hochberg and |logFC| > 0.5) upregulated genes in fetuses and embryos are highlighted in red (1) and blue (−1), respectively, and non-differentially expressed genes are highlighted in black (0).

In DE analysis, a total of 1705 genes were differentially regulated (adjusted *p*-value < 0.05 and |logFC| > 0.5) between pig fetuses and embryos, in which 1258 genes were upregulated in pig fetuses only, and 447 genes were upregulated in pig embryos (Figure 2). The top 10 genes upregulated in pig embryos and the top 10 genes upregulated in pig fetuses according to the logFC (Table 1) were related to erythrocyte differentiation, heart development, ions transport, skeletal muscle system, neuronal development, ossification, and renal function. The complete list containing all DE genes is available in Table S1.

### 3.3. Functional Analyses

**Figure 3:**
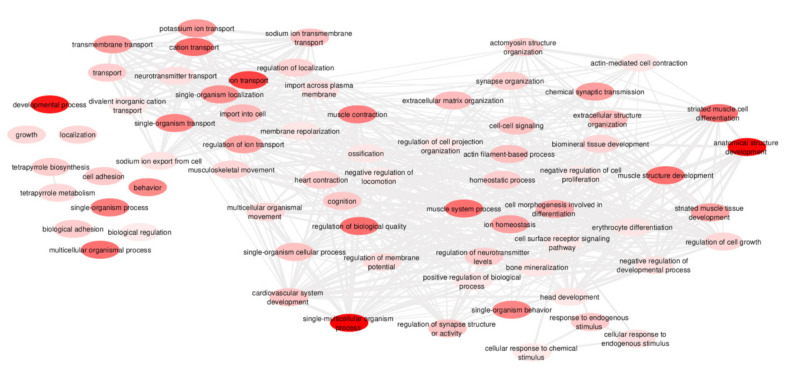
Enriched biological processes (bubbles) (adjusted *p*-value < 0.05 from Benjamini–Hochberg) obtained in the REVIGO tool for all differentially expressed genes between pig fetuses and embryos. Bubbles´ color represents the adjusted *p*-value of the gene ontology (GO) terms, in which a more intense red color indicates more enriched biological processes. Line width indicates the degree of similarity among GO terms, in which thinner lines represent less similarity among GO terms. The design of this network was modified in Cytoscape version 3.8.0.

In GO analysis, a total of 203 GO terms associated with BP categories were enriched (FDR < 0.05) using the DAVID tool and submitted to the REVIGO tool, resulting in 71 BP (Figure 3). The enriched GO terms highlighted several BP crucial for developmental biology, such as anatomical structure development, regulation of cell growth, negative regulation of development process, and regulation of biologic quality. Additionally, other BP related to the physiology of embryonic to fetal transition, including ossification, extracellular matrix organization, skeletal muscle development, erythrocyte differentiation, neuronal system, and cardiovascular system, were enriched by many of the top DE genes between pig fetuses and embryos. More detailed information regarding the GO terms is available in Table S2.

**Figure 4:**
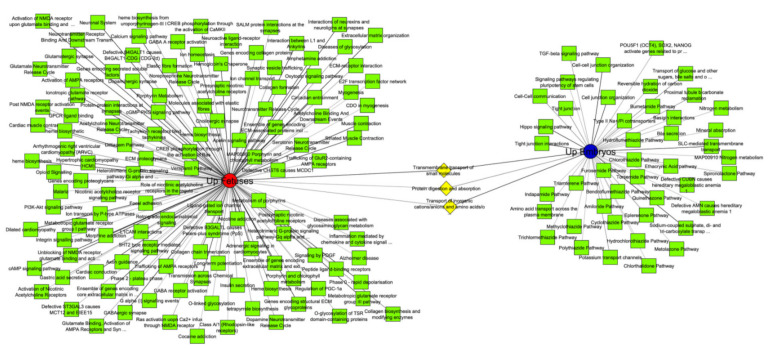
Pathway network for annotated genes upregulated in pig embryos and fetuses. The upregulated genes in pig embryos (blue) and in fetuses (red) were submitted to the ToppCluster online tool. Pathway category (adjusted *p*-value < 0.05 from Benjamini–Hochberg) was used in functional enrichment and Cytoscape version 3.8.0 was used to modify the network design. Green nodes represent specific pathways and yellow nodes represent shared pathways between embryos and fetuses.

From the 173 pathways obtained in the Toppcluster tool, 44 specific pathways were enriched by upregulated genes in pig embryos. Among them, we found genes related to Hippo and TGFB signaling pathways, pluripotency, and proliferation pathways, as well as cell–cell interaction (Figure 4). Other pathways were enriched by upregulated genes in pig fetuses, including myogenesis, collagen synthesis, and neuronal systems pathways (Figure 4). These pathways are in line with the greater number of differentiated tissues established at the fetal development compared to embryonic stage. Moreover, we detected three pathways shared between upregulated genes in both embryos and fetuses, mostly related to transport of small molecules, transport of anions/cations, and protein digestion and absorption. These enriched pathways were encoded by genes related to transporters proteins, such as the Solute Carrier family and ATP-binding pumps, and collagen compounds, including the *COL6A6* that was one of the most upregulated genes in pig fetuses. Additional details regarding the pathways enriched for the embryos and fetuses’ development, as well as the pathways shared by them, are available in Table S3.

## 4. Discussion

Organogenesis is a crucial period in which the main tissues, organs, and systems are established [2,26]. During organs development, a tight transcriptional control of genes involved in cell-cell interaction, and cellular proliferation and differentiation is required to ensure the normal development and functionality of the organs [27]. In this study, through RNA-seq analysis, we identified several DE genes that enriched important BP during pig prenatal development, such as biological regulation of cell proliferation and differentiation, cell morphogenesis, growth, and ossification. Moreover, the general pathways of developmental biology, such as Hippo and morphogenic related pathways, E2F transcription factor and more specialized pathways, including myogenesis, neurodevelopmental and extracellular matrix assembly, were evidenced from DE genes. Since these events are determinant for the successful establishment of tissues, organs, and body structures, the complete organogenesis overview provided by this study can contribute to further studies addressing specific biological events during prenatal development.

### 4.1. General Pathways during Prenatal Development

The Hippo pathway was initially discovered during organs growing in *Drosophila*, and it is highly conserved in mammals, mediating transcriptional control during the development, homeostasis, and pathological conditions [27,28,29]. In the developmental context, in this study, the Hippo signaling pathway was enriched by upregulated genes in pig embryos, including genes that encode the Hippo pathway upstream modulators, the FERM Domain Containing 1 (*FRMD1*), Crumbs Cell Polarity Complex Component 2 (*CRB2*), LGL/LLGL Scribble Cell Polarity Complex Component 2 (*LLGL2*), Par-6 Family Cell Polarity Regulator Beta (*PARD6B*; member of PAR6 family), Membrane Palmitoylated Protein 5 (*PALS1*/*MPP5*), PALS1-Associated Tight Junction Protein (*PATJ*), Ras Association Domain Family Member 6 (*RASSF6*), and Ajuba LIM Protein (*AJUBA*) [27,30]. Although the mechanism and critical physiologic functions of these upstream proteins in mammalian Hippo signaling are not completely clear, some of them are also components of cell-cell junctions (CRB1-3, PAR6, PALS1 and AJUBA) [27], suggesting that the Hippo signaling is regulated by multiple inputs, including cell polarity and density, cytoskeleton, and extracellular environment interaction during the development [27,29]. Accordingly, the *CRB2*, *MMP5*, and *PARD6B* genes related to Hippo signaling were also associated with tight junction and cell junction pathways enriched for embryos development. The multiple inputs modulate the activity of Hippo signaling downstream effectors, such as YAP (Yes1 Associated Transcriptional Regulator; *YAP1*), which bind to transcription factors in the nucleus and promote the expression of target genes related to cell proliferation, differentiation and apoptosis [27]. The encoding *YAP1* gene was also upregulated in pig embryos and associated with Hippo pathway, which may reflect its association with transcriptional control during early events related to organogenesis in pigs, since the YAP has been associated with brain, heart, kidney, liver and lung development (for review, see [29,31]), and cell fate determination in preimplantation embryos [32,33]. Therefore, our findings reinforce the transcriptional regulation mediated by the Hippo signaling pathway mainly during pig embryonic stage, since multiple inputs from cell-cell interaction and extracellular environment are crucial for organogenesis related events, such as cell fate and cell differentiation, mainly during embryogenesis [29].

Despite the key role of Hippo signaling in transcriptional control, a possible crosstalk between Hippo and other signaling pathways, such as TGFB (Transforming Growth Factor Beta), can be required to enhance the transcriptional responses during critical stages of developmental biology [28,30]. In this sense, TGFB signaling pathway was enriched by upregulated genes in pig embryos here, in which *TGFBR1* (Transforming Growth Factor Beta Receptor type 1) and *BMP4* (Bone Morphogenetic Protein 4) genes were also associated with Hippo signaling pathway. The TGFB signaling is required for cell patterning, organogenesis, and tissue homeostasis from binding of TGFB superfamily members, including TGFBs and BMPs, to transmembrane receptors type I and II [34,35,36]. The BAMBI (BMP and Activin Membrane Bound Inhibitor; BAMBI) is a transmembrane protein structurally similar to receptors type I of TGFB family, except by the kinase activity required for signaling [35]. Since BAMBI dimerizes with I and II receptors of TGFB superfamily, inhibiting the transcriptional response mediated by TGFBs and BMPs, the BAMBI is related to developmental homeostasis [37,38]. Our findings showed that the *BAMBI* gene was upregulated in pig embryos, being associated with TGFB signaling pathway. Additionally, *BAMBI*, *TGFBR1*, and *BMP4* genes shared several BP, such as development processes, cell surface receptor signaling, cellular response to stimulus, and cell morphogenesis involved in differentiation, biological regulation, positive regulation of biological processes, and anatomical structure. Taken together, our findings highlight important mediators of the TGFB superfamily signaling, which may be relevant for developmental homeostasis during pig organogenesis. This fact is mainly relevant for the embryonic stage, once the morphogenesis of the heart, limb, and gonads are events particularly sensitive to the TGFB superfamily signaling [39].

As long as the prenatal development advances, a greater degree of cell specialization is required for normal tissue functionality, depending on a strict control of cell proliferation and differentiation [40,41] which can be mediated by E2F family of transcription factors and their regulators (for review, see [41]). In line with this, the E2F transcription factor network was enriched by *E2F2*, *RB1*, *CCND3* (D3-type cyclin), *CCNE2* (E2- type cyclin) genes and cyclin-CDK complex regulators, namely *CDKN1A* (Cyclin Dependent Kinase Inhibitor 1A, encoding a component of Cip/Kip inhibitor family) and *CDKN2C* (Cyclin Dependent Kinase Inhibitor 2C, encoding a component of INK4 inhibitor family) genes that were upregulated in pig fetuses. In addition, the *RB1*, *CDKN2C* and *CCNE2* genes enriched BP related to cell differentiation, such as biological regulation, negative regulation of cell proliferation, striated muscle cell differentiation and erythrocyte differentiation. Since the cell cycle control is required to avoid developmental abnormalities mainly in differentiated tissues [40] and the transcriptional control involving E2F pathway is associated with pluripotent cells differentiation [42], the upregulation of genes related to E2F pathway in pig fetuses might evidence a transcriptional regulation to maintain the differentiated cells, which is required for a proper tissue and organ development and function.

Furthermore, the *KAT2B* gene was also associated with E2F transcription factor network enriched for pig fetuses’ development, and shared with *RB1* and *CDKN2C* the biological regulation and negative regulation of cell proliferation BP. The *KAT2B* gene encodes the p300/CBP-associated factor containing a histone acetyl transferase (HAT) domain, which has also been associated with acetylation of non-histone proteins [43], such as E2F1, and forms a pro-apoptotic complex with RB and E2F1 [44]. Moreover, recently, the *KAT2B* gene has been related to cell growth and differentiation during craniofacial and cartilage development in zebrafish and mouse embryos, which may be mediated by acetylation activity of the encoded protein [45]. These findings reinforce important levels of gene transcription control related to proliferation and differentiation mediated by E2F transcription factors, as well as their partners, which is in line with the control of cell cycle progression to maintain the function and development of specialized organs and systems that are more numerous at fetal stage, as highlighted in further sections of this article.

### 4.2. Cardiovascular System

Among the events related to this system is the establishment of the heart, which is a primitive tube around 21-22 days of gestation [1], and thus, undergoes morphogenic changes to four-chambers based-system (left and right ventricles and atrium) [46,47]. In this context, the *HAND1* gene that encodes the transcription factor HAND1 (Heart And Neural Crest Derivatives Expressed 1), which is exclusively expressed in left ventricle during heart development and induces the expression of cardiac genes [1,48], was one of the most upregulated genes in pig embryos at 25 days-old. Similarly, the chamber septation is visible in mammalian species at similar gestational age, including equine embryos at 25 days-old [49], dog and cat embryos around 23-25 days-old [3]. The heart septation allows the pumping of blood based on routes instead of unidirectional flow, which is required due to the quick fetal growth [47]. Therefore, the upregulation of *HAND1* gene in pig embryos suggests the heart morphogenesis and induction of cardiac genes expression, which are important events to ensure the satisfactory supply of nutrients and oxygen for the development of many tissues and organs that will continue to grow at fetal stage. Additionally, this upregulation of *HAND1* gene in pig embryos reinforces its essentiality for normal cardiac system development at early prenatal life, since deletion of this gene in mouse embryos resulted in cardiac abnormalities, including ventricular malformation and dysregulated cardiac gene expression, leading to death in the perinatal stage [50].

Once stablished, the heart presents fast and irregular contractions that start around 21-22 days of gestation in mammalian species [1,51,52]. However, in humans, the first expressive increase in the heart contraction is detected from embryo to fetus transition, as a consequence of increased heart size and circulatory system maturation, which are events necessary to ensure adequate blood supply for body growth [51,53]. In line with the greater activity of the heart in fetal stage, the cardiac conduction pathway was specifically enriched by upregulated genes in pig fetuses, including genes related to contractile activity, such as ATPases (*ATP1A2*, *ATP1B2*, *ATP2A1*, *ATP2B4*), Calcium Voltage-Gated Channel (*CACNA1D*, *CACNA1S*, *CACNA2D1*, *CACNA2D2*, *CACNA2D3*, *CACNB1*, *CACNG1*, *CACNG5*), Ryanodine Receptor 3 (*RYR3*), Alpha Actin Cardiac Isoform (*ACTC1*) and Troponin Cardiac Isoforms (*TNNC1*, *TNNI1*). Furthermore, some of these genes, including *TNNC1*, *TNNT2* and *ACTC1*, enriched the Hypertrophic cardiomyopathy (HCM) pathway. The HCM is characterized as a pathological condition of cardiac contraction caused by defects in the sarcomeric proteins, which result in failure of heart contraction as a primary defect, followed by cardiac cells hypertrophy as a compensation mechanism for this stress [54]. The enrichment of pathways related to cardiac conduction by upregulated genes during pig fetal development highlights the cardiac contraction activity required for the faster body growth in the fetal stage.

During the cardiovascular system development, the primitive vascular plexus and blood vessels are formed, which can be mediated by signaling molecules including Semaphorin [46]. Regarding this, the cardiovascular system BP was enriched by members of Semaphorin family, such as *SEMA3E* (Semaphorin 3E) gene, which was upregulated in pig embryos, and *SEMA3C* (Semaphorin 3C) gene, upregulated in fetuses. The *SEMA3E* gene is associated with vascular patterning (vasculogenesis), in which the blood vessels are originated from endothelial progenitor cells (EPCs) or angioblasts [55], whereas the *SEMA3C* gene has been associated with angiogenesis, defined as new blood vessels formation from preexistent vessels (for review, see [46]). Moreover, the Notch signaling pathway is required for vessels formation, stabilization, homeostasis and anastomosis, while the Angiopoietin 2 (ANGPT2) is responsive to angiogenic stimulus, which destabilizes the quiescent vasculature and activates the endothelial cells differentiation to form new vessels [56]. The cardiovascular system was also enriched by *NRARP* (Notch-Regulated Ankyrin Repeat Protein) gene, upregulated in pig embryos, and *ANGPT2* gene, upregulated in fetuses. The upregulation of genes related to vasculogenesis (*SEMA3E*) and vessels stabilization (*NRARP*) in pig embryos and related to angiogenesis (*SEMA3C* and *ANGPT2*) in fetuses suggests a differential regulation of the blood vessels formation during prenatal development. This is in line with the importance of vasculogenesis to form the main vessels (dorsal aorta and cardinal vein) and primitive vascular plexus mainly at embryonic stage [46,47,57], and all the other blood vessels from angiogenic stimulus [47] at fetal stage, which is mediated by angiogenic genes.

The circulatory system development also involves the erythrocytes maturation, which are the first cells identified as blood cells during prenatal development and undergo many stages of differentiation until became mature and definitive blood cells [58]. The primitive erythrocytes, produced in yolk sac, mainly express genes of the globin Epsilon (*HBE1*), a member of beta like globin, and Zeta (*HBZ*) subunits, a member of alpha like globin, known as embryonic globin genes, while the definitive erythroid cells, produced in the fetal liver, mainly express fetal globin genes of Gamma (*HBG*) and adult globin subunits, such as Beta (*HBB*) [59,60]. Considering the tight transcriptional control of globin genes during the development, these genes that enriched BP related to blood cells, including erythrocyte differentiation and transport, were the most differentially regulated in prenatal pig transcriptome, being the *HBZ* and *HBE1* genes the most upregulated genes in embryos, while the *HBB* gene was one of the most upregulated gene in fetuses. The switch from primitive to definitive erythrocytes mediated by globin gene expression is compatible with the quick fetal growth, since as the prenatal development advances, more oxygen needs to be extracted from maternal blood, which is supported by enucleated definitive erythrocytes [58]. In addition to globin genes, the erythrocytes maturation involves the erythropoietin synthesis encoded by *EPO* gene required for primitive erythroid cells maturation (for review, see [60]) and onset of definitive erythropoiesis in mouse embryos [61], and GATA1 (GATA Binding Protein 1; *GATA1*) and KLF1 (Kruppel Like Factor 1; *KLF1*) transcription factors are involved in transcription of genes related to definitive erythropoiesis (for reviews, see [60,62]). In line with primitive erythropoiesis, the *EPO* gene was upregulated in pig embryos, while *GATA1* and *KLF1* genes related to definitive erythropoiesis were upregulated in pig fetuses.

### 4.3. Kidney Development

The mammalian kidney development involves transition of the three main structures, the pronephros, mesonephros (intermediary kidney), and metanephros (definitive kidney) [49,63]. A temporal overlapping mainly related to mesonephros and metanephros development is observed in several mammalian species, including pigs, in which the mesonephros structure is observed from 15 to 24 days of gestation, overlapping with metanephros developing structure around 20 days of gestation [64]. Regarding this gestational period, genes related to kidney organogenesis were upregulated in pig embryos at 25 days old. Among them are genes encoding key transcription factors required for mesonephric tubules development, renal vesicles formation, and metanephros function, such as SALL1 (Spalt Like Transcription Factor 1; *SALL1*), and WT1 (Wilms Tumor Protein; *WT1*) [65,66], as well as morphogens inductors, such as WNT9B (Wnt Family Member 9B; *WNT9B*) [65,67], associated with mesonephric kidney tubules and renal vesicle formation, and initial induction of nephron development from morphogenic stimulus [65,66,67]. In the functional analyses, *SALL1* and *WT1* genes shared the anatomical structure, biological regulation, and cell morphogenesis BP, and the *WNT9B* gene was associated with the regulating pluripotency of stem cells signaling pathway enriched for embryonic stage, which are events that can be associated with initial signaling for kidney organogenesis.

The nephron, which is a common filtering unit among stages of kidney organogenesis, is mainly composed by glomerulus, proximal and distal ducts at early stages of kidney development [68]. The glomerulus is the most complex filtering structure, and podocytes are the main specialized cells that maintain its function [67,69]. There are many genes expressed by glomerular podocytes during kidney development, including the above mentioned *WT1* gene, and *NPHS1* (Nephrin), and *NPHS2* (Podocin) [1,67,70], which were also upregulated in pig embryos in the current study. Moreover, genes members of Solute Carrier family (SLC), aquaporins and carbonic anhydrase [70], were upregulated in pig embryos and are related to the physiologic function of renal structures. Additionally, the *SLC9A2* and *SLC39A2* genes were expressed in the collecting duct in human kidney development, whereas *SLC3A1* and *SLC34A3* genes were expressed in proximal duct [70]. In addition, members of Aquaporins (*AQP3*) and Carbonic Anhydrase (*CA2*), which are involved in water reabsorption and acid-basic equilibrium and were also considered as markers of collecting and proximal ducts in human prenatal kidney development [70], were upregulated in pig embryos. During embryonic development of many species including pigs and humans, the mesonephros are considered highly active structures [64,71], which are well-developed in 25 day-old pig embryos [71]. Accordingly, the functional markers of glomeruli filtering (*NPHS1* and *NPHS2* genes), and distal and proximal tubules (SLC gene members, *AQP3* and *CA2* genes) enriched pathways related to renal function, such as nitrogen metabolism, mineral absorption, and proximal tubule bicarbonate reclamation during embryonic development. Taken together, our findings may indicate the transcriptional control compatible with the development and functionality of mesonephros during pig embryogenesis. Moreover, these findings provide valuable information for further investigation about this intermediary structure, since only recently the prenatal stages of kidney development have been reported in pigs [71] and many of the current knowledge regarding kidney organogenesis at molecular level is based on metanephros [65,72].

### 4.4. Skeletal Muscle Development

Myogenesis is the most well-described biological event during the prenatal development in livestock, since the number of muscle fibers is determined in the fetal stage and it is closely related to muscle mass [73,74]. Among genes that encode Muscle Regulatory Factors (MRFs), the *MYF5* (Myogenic Factor 5) and *MYOD1* (Myogenic Differentiation 1), which regulate myoblasts differentiation from myogenic precursor, and *MYF6* (Myogenic Factor 6) and *MYOG* (Myogenin), which are markers of myotubes formation from myoblasts fusion [73,75,76], were upregulated in pig fetuses at 35 days-old. These findings are in line with the formation of the primary myotubes during first myogenic wave [76], that occurs around 35-55 days of gestation in pigs [77]. Accordingly, genes expressed by primary myotubes [76], such as *MYH3* (Myosin Heavy Chain embryonic) and *MYH8* (Myosin fetal/neonatal isoforms), were also upregulated in pig fetuses.

During myogenesis, the Myostatin (*MSTN*) is an important negative regulator of myoblasts proliferation, stimulating the muscle fiber differentiation [78], and the Myomaker Myoblast Fusion Factor (*MYMK*/*TMEM8C*) is a key muscle-specific factor required for myoblast fusion [79]. These encoding genes were upregulated in pig fetuses and shared many BP, including developmental process, homeostatic process, muscle structure development and anatomical structure, reflecting their physiological role during muscle development. These findings highlight events required for normal skeletal muscle development, which are mainly important during fetal development, when myoblasts proliferate and fuse.

The molecular mechanism that culminates in muscle growth is mediated by the mitogenic factors, such as IGFs (Insulin-like Growth Factors), that binds to IGF1R (Insulin-like Growth Factor 1 Receptor) [80,81]. The availability of IGFs is regulated by IGFBPs (Insulin Growth Factors Binding Proteins), which may result in increased or reduced IGF release for binding to its receptor [81]. Particularly, during myogenesis, greater levels of IGBP-5 are needed for myoblasts differentiation, since the increase in the IGFBP-5 levels increases the local concentration of the IGF-2 ligand, increasing its release to IGFR1 (for review, see [81]). The *IGFBP-5* gene, that was also upregulated in pig fetuses, shared with MRFs genes BP that may be associated with myogenesis, including response to endogenous stimulus, growth and biological regulation BPs. Additionally, based on *IGFBP5* and other IGFBP family genes, including *IGFBP4*, *IGFBP6* and *IGFBP7*, which were upregulated in pig fetuses, and the lack of differentially regulated IGFs genes, it is suggested that IGFBPs rather than IGF itself may act as major molecular mediators of growth during pig prenatal development.

### 4.5. Skeleton Development

The skeleton development occurs simultaneously with myogenesis, given the complementary functional properties of the musculoskeletal system, such as body stability [82]. During embryogenesis, the body patterning in three major skeletons (craniofacial, axial and appendicular) is mediated by transcription factors of Hox family and signaling molecules, such as SHH (Sonic Hedgehog) (for review, see [83]). In the pig prenatal transcriptome, *SHH* gene and the Hox family genes (*BARX1*, *MSX1*, *HOXA1*, *HOXA2*) were upregulated in pig embryos and enriched the BP related to body pattern, such as head development (*SHH*, *MSX1* and *HOXA2* genes) and anatomical structure development (*SHH*, *HOXA1*, *HOXA2*, *BARX1* and *MSX1* genes). In addition, the *TWIST1*/*TWIST* (Twist Family BHLH Transcription Factor 1) and *DLL3* (Delta Like Canonical Notch Ligand 3) genes, which have previously been associated with skeletal patterning [83], were also upregulated in pig embryos. Since these genes induce specific transcriptional programs necessary for cell patterning events, mainly important in embryonic development [1,83], these findings are in accordance with the body patterning from transcription factors and signaling molecules at early prenatal development.

As the muscle skeletal system is established, the initial signals for bone development are required, which is observed in early equine fetuses [49]. The bone matrix synthesis depends on osteoblasts, that differentiate from the mesenchymal progenitor cells [83] in a process that can be mediated by WNT signaling stimulating pro-osteogenic transcription factors, such as OSX/SP7 (Osterix/Sp7 Transcription Factor) [84]. In the present study, members of the WNT signaling, namely *WNT9A*/*WNT14* (Wnt Family Member 9A/14), *FDZ9* and *LRP5* genes, as well as *SP7* gene, were upregulated in pig fetuses. Moreover, the *WNT9A* and *FZD9* genes shared the cell surface receptor and anatomical structure development BP. Since the activation of the Frizzled receptor (FZD) and/or the Lipo-Protein Receptor-Related Protein (LRP5/LRP6) by WNT molecules binding, such as WNT9A/WNT14 [85,86], leads to beta-catenin stabilization and the expression of pro-osteogenic factors, including OSX/SP7 [84,87], our findings may provide an important molecular mechanism involved in osteoblasts differentiation in pig fetuses.

Upon differentiation, the osteoblasts and other cells, as odontoblasts, start to express genes that encode proteins involved in extracellular matrix mineralization [83,88]. Several of these genes were upregulated in fetuses, including Osteopontin, also known as Secreted Phosphoprotein 1 (*SPP1*), Bone Sialoprotein (*IBSP*/*BSP*), which was one of the most upregulated genes in pig fetuses, Osteomodulin (*OMD*), Dentin Matrix Acidic Phosphoprotein-1 (*DMP1*) and Phosphate-Regulating Endopeptidases Homolog X-Linked (*PHEX*) [87,88,89,90,91]. Additionally, these genes were associated with ossification, biomineral tissue development and bone mineralization BP. In line with osteoblasts differentiation during pig fetal development, the upregulation of genes related to bone development in pig fetuses highlights initial signals required for bone development and mineralization, which are important events related to skeleton development and embryonic-fetal transition in pigs [92].

### 4.6. Extracellular Matrix and Prenatal Development

The extracellular matrix (ECM) is copiously synthetized by differentiated cells to maintain the mechanical and biomechanical features of the specialized tissue, including bone and muscle [93,94]. The collagen is the most abundant component of the ECM, and it is widely distributed among body tissues [93]. In the pig prenatal transcriptome, several coding genes for collagen compounds were upregulated in fetuses and enriched collagen biosynthesis related pathways. Among these genes, the members of collagens type VI, such as *COL6A2*, *COL6A3* and *COL6A6*, the latter being one of the most upregulated genes in pig fetuses, are important to maintain the skeletal muscle functional integrity [95]. Therefore, the upregulation of these genes at fetal stage is in line with the myogenesis related events, which were more evident in pig fetuses from upregulation of MRFs genes.

Among the non-collagen compounds of the ECM are Matrilin 1 (*Crtm*/*MATN1*) and Aggrecan (*Agc1*/*ACAN*), which are synthesized and secreted by chondrocytes and highly active chondroblasts during prenatal development [96]. In equine embryos at 35 days of gestation, the chondrocytes and chondroblasts are present in the bud of limbs and in some developing vertebrate [49]. Therefore, the upregulation of the *MATN1* and *ACAN* genes in pig fetuses may suggest increased synthesis of cartilaginous tissue at this development stage. The cartilaginous tissue, besides contributing to the biomechanical stability of the organs, is crucial during endochondral ossification, which is when this abundantly secreted tissue is remodeled to allow the invasion of cells that will act in the ossification process, including osteoblasts [96,97,98]. The matrix Metalloproteinases (MMPs) and the Tissue Inhibitors of Metalloproteinases (TIMPS) are associated with ECM remodeling during endochondral ossification (for review, see [97,99]). In our study, the *MMP2* and *TIMP2* genes were upregulated in fetuses. According to their function during endochondral ossification, the *MMP2* gene was associated with ossification BP, while the biological events potentially involved in the modulation of its proteolytic activity, such as response to endogenous stimulus and biological regulation, were enriched by *TIMP2* gene. Taken together, these findings highlight the function of these genes during bone formation in pig fetuses at 35 days-old of gestation, through ECM remodeling.

### 4.7. Neuronal Development

The neurogenesis is a biological event in which the stem cells or progenitor cells differentiate into neurons [100]. During neurogenesis, the neural basic helix-loop-helix (bHLH) transcription factors, which induce the neurogenesis transcriptional program and activation of the neuronal differentiation effectors [101], are differentially expressed in order to ensure the correct neuronal identity during prenatal development [102,103]. In this context, the members of neural bHLH were differentially regulated in pig prenatal transcriptome, namely *NEUROG1* (Neurogenin 1) gene, upregulated in pig embryos, and *NEUROD6* (Neuronal Differentiation 6) gene, which was one of the most upregulated genes in pig fetuses. This differential expression reflects the functional role of *NEUROG1* during embryogenesis, which acts as a cell fate determination factor and is exclusively expressed by neuronal proliferative cells [103], while the *NEUROD6* is exclusively expressed by differentiated neurons [102]. Therefore, the upregulation of *NEUROG1* gene in pig embryos suggests an initial transcriptional program required for neuronal differentiation during embryogenesis, which was also evidenced by its association with signaling pathways regulating pluripotency of stem cells pathway, whereas the upregulation of the *NEUROD6* gene in pig fetuses highlights the presence of differentiated neuronal cells.

During neuronal development, the excitatory neurons migrate from the proliferative zones to the cerebral cortex surface, characterizing the radial migration, while in the tangential migration, inhibitory neurons migrate from ganglionic eminence also toward cortical region [104]. The glutamate receptors, including Glutamate Ionotropic Receptor AMPA type, and GABA receptors, including Gamma-Aminobutyric Acid Type A Receptor, are involved in the neuronal migration during brain prenatal development [105,106,107]. In the current study, many genes encoding different subunits of the glutamatergic AMPA type receptors, such as *GRIA1*, *GRIA2*, *GRIA3*, *GRIA4*, and GABAergic type A receptors, such as *GABRA3*, *GABRA4*, *GABRA5*, *GABRB1*, *GABRB2*, *GABRB3* and *GABRG2*, were upregulated in pig fetuses and enriched the neuronal system pathway. In addition to these receptors, the gene encoding Roundabout Guidance Receptor 3 (*ROBO3*), which has also been related to cortex development and neuronal migration [108], was also upregulated in pig fetuses. In humans, an intense neuronal migration is observed at fetal stage in order to create a population of cortical neurons [109]. Our results suggest important signals for neuronal migration in pig fetal development in line with events related to neurodevelopment that were also highlighted at this stage, including synaptogenesis.

The synaptic transmission is dependent on the suitable balance between excitatory and inhibitory neurons in the cortex [104,110]. In humans, the synaptogenesis starts from the embryos to fetuses transition as transitory circuits, which are important to ensure normal brain development and function [109,111]. In line with this early synaptogenesis in humans, several upregulated genes in pig fetuses were associated with synapse organization, regulation of synapse structure, regulation of synapses activity and chemical synapses transmission BP, including, in addition to GABAergic and glutamatergic receptors encoding genes, genes that encode molecules directly involved in the synaptogenesis, such as *SYT1* (Synaptotagmin 1), *SYN1* (Synapsin I), *SYP* (Synaptophysin), *LRRTMs* (Leucine Rich Repeat Transmembrane proteins) and *NRXN1* (Neurexin 1) [105,110]. These findings highlight genes that encode integral proteins of synaptic vesicles, which may be related to synaptic transmission events during pig fetal development. On the other hand, in mouse, the synthesis of synaptogenic molecules, including SYT1, SYN1 and SYP, and the first synapses only occur in postnatal life [110,112]. Since the early prenatal synaptogenesis is related to greater complexity of the neurodevelopment [112], our findings, besides suggesting that the transcriptional control of synaptogenesis is an event required for proper neurodevelopment at fetal stage, reinforce the similarities between pig and human fetuses regarding neuronal development, which can support further studies addressing human prenatal issues related to synaptic plasticity, such as learning and memory [105].

## 5. Conclusions

This study provides a comprehensive and exploratory landscape about the transcriptional changes associated with morphogenesis and organogenesis in pigs. During the embryonic stage, genes related to Hippo signaling and TGFB/BMP4 signaling pathway evidenced the transcriptional regulation from cell interactions and morphogens, which coordinate the transcriptional control mainly at early prenatal development. On the other hand, biological processes and pathways related to myogenesis, neuronal development, and cardiac and striated muscle contraction were evidenced mainly by upregulated genes in fetuses, reflecting the greater complexity of organs and body structures at this stage of development. Therefore, in the current study we describe the genes and networks that shall be further disentangled in future studies addressing more specific biological events, not only in the pig but also in other mammals.

## Figures and Tables

**Table 1:** Top 10 upregulated genes in pig embryos and top 10 upregulated genes in pig fetuses.

Embryos				
Ensembl ID	logFC ^1^	Gene Name	Gene Description	adj. *p*-Value ^2^
ENSSSCG00000014726	−6.764396328	*HBE1*	Hemoglobin subunit epsilon 1	1.38 × 10^−8^
ENSSSCG00000007975	−6.379504386	*HBZ*	Hemoglobin subunit zeta	8.50 × 10^−10^
ENSSSCG00000031865	−5.217417343	*AQP8*	Aquaporin 8	8.04 × 10^−6^
ENSSSCG00000021902	−4.96041836	*GABRP*	Gamma-aminobutyric acid type A receptor subunit pi	3.70 × 10^−12^
ENSSSCG00000040513	−4.537759202	*AQP3*	Aquaporin 3	1.18 × 10^−9^
ENSSSCG00000006731	−4.451641793	*VTCN1*	V-set domain containing T cell activation inhibitor 1	7.08 × 10^−10^
ENSSSCG00000031080	−3.795925653	*HAND1*	Heart and neural crest derivatives expressed 1	6.49 × 10^−7^
ENSSSCG00000000418	−3.699759861	*TAC3*	Tachykinin precursor 3	1.53 × 10^−11^
ENSSSCG00000030461	−3.607432588	*HEPHL1*	Hephaestin like 1	2.04 × 10^−9^
ENSSSCG00000002432	−3.418006247	*KCNK13*	Potassium two pore domain channelsubfamily K member 13	1.87 × 10^−8^
Fetuses				
ENSSSCG00000009219	7.553512845	*IBSP*	Integrin binding sialoprotein	3.58 × 10^−12^
ENSSSCG00000014725	7.383041924	*HBB*	Hemoglobin, beta	1.01 × 10^−10^
ENSSSCG00000040098	6.688192867		Uncharacterized	1.21 × 10^−11^
ENSSSCG00000037430	6.022006128	*COL6A6*	Collagen type VI alpha 6 chain	3.09 × 10^−10^
ENSSSCG00000039501	6.020672325	*NEUROD6*	Neuronal differentiation 6	4.32 × 10^−8^
ENSSSCG00000008072	5.978854537	*ASPN*	Asporin	1.58 × 10^−14^
ENSSSCG00000009955	5.824620103	*CRYBB2*	Crystallin beta A2	9.52 × 10^−8^
ENSSSCG00000035520	5.774356588		Uncharacterized	0.01103
ENSSSCG00000040641	5.70621141	*CRYBA1*	Crystallin beta A1	9.23 × 10^−5^
ENSSSCG00000031903	5.684525698	*TNNT3*	Troponin T3, fast skeletal type	2.33 × 10^−12^

^1^ Log2-fold change values through contrasts between pig fetuses and embryos; ^2^ Adjusted *p*-value for multiple correction testes to reduce type I error; significance threshold: Adjusted *p*-value < 0.05 and |logFC| > 0.5.

## Data Availability

Publicly available datasets were analyzed in this study. This data can be found here: https://www.ncbi.nlm.nih.gov/sra/?term=PRJNA576701, accessed on 10 October 2019.

## Supplementary Materials

The following are available online at https://www.mdpi.com/article/10.3390/ani11051443/s1, File S1: Materials and Methods—additional information, Table S1: DE genes. Table S2: CPM_DE. Table S3: logCPM_DE genes. Table S4: Biological Processes. Table S5: Pathways.

## References

[1] Hyttel P., Sinowatz F., Vejlsted M., Betteridge K., Edwards R., Rodenhuis J., Betteridge K. (2010). Essentials of Domestic Animal Embriology.

[2] Edwards M.J., Saunders R.D., Shiota K. (2003). Effects of heat on embryos and foetuses. Int. J. Hyperth..

[3] Pieri N.C.G., Souza A.F., Casals J.B., Roballo K.C.S., Ambrósio C.E., Martins D.S. (2015). Comparative Development of Embryonic Age by Organogenesis in Domestic Dogs and Cats. Reprod. Domest. Anim..

[4] Danesh S.M., Villasenor A., Chong D., Soukup C., Cleaver O. (2009). BMP and BMP receptor expression during murine organogenesis. Gene Expr. Patterns.

[5] Wesolowski S.R., Raney N.E., Ernst C.W. (2004). Developmental changes in the fetal pig transcriptome. Physiol. Genom..

[6] Foxcroft G.R., Dixon W.T., Novak S., Putman C.T., Town S.C., Vinsky M.D. (2006). The biological basis for prenatal programming of postnatal performance in pigs. J. Anim. Sci..

[7] Ji Y., Wu Z., Dai Z., Wang X., Li J., Wang B., Wu G. (2017). Fetal and neonatal programming of postnatal growth and feed efficiency in swine. J. Anim. Sci. Biotechnol..

[8] Lin H., Wang H., Wang Y., Liu C., Wang C., Guo J. (2015). Transcriptomic Analysis of the Porcine Endometrium during Embryo Implantation. Genes (Basel).

[9] Zeng S., Ulbrich S.E., Bauersachs S. (2019). Spatial organization of endometrial gene expression at the onset of embryo attachment in pigs. BMC Genom..

[10] Te Pas M.F.W., De Wit A.A.W., Priem J., Cagnazzo M., Davoli R., Russo V., Pool M.H. (2005). Transcriptome expression profiles in prenatal pigs in relation to myogenesis. J. Muscle Res. Cell Motil..

[11] Sollero B.P., Guimarães S.E.F., Rilington V.D., Tempelman R.J., Raney N.E., Steibel J.P., Guimarães J.D., Lopes P.S., Lopes M.S., Ernst C.W. (2011). Transcriptional profiling during foetal skeletal muscle development of Piau and Yorkshire-Landrace cross-bred pigs. Anim. Genet..

[12] Costa K.A., Saraiva A., Guimaraes J.D., Marques D.B.D., Machado-neves M., Reis L.M.B., Alberto F., Veroneze R., Oliveira L.F.D., Garcia I.S. (2019). Dietary L-arginine supplementation during early gestation of gilts affects conceptuses development. Theriogenology.

[13] Garcia I.S., Teixeira S.A., Costa K.A., Marques D.B.D., Rodrigues G.d.A., Costa T.C., Guimarães J.D., Otto P.I., Saraiva A., Ibelli A.M.G. (2020). l- Arginine supplementation of gilts during early gestation modulates energy sensitive pathways in pig conceptuses. Mol. Reprod. Dev..

[14] Teixeira S.A., Ibelli A.M.G., Cantão E., Oliveira H.C.D., Ledur M.C., Peixoto J.d.O., Marques D.B.D., Costa K.A., Coutinho L.L., Guimarães S.E.F. (2019). Sex Determination Using RNA-Sequencing Analyses in Early Prenatal Pig Development. Genes (Basel).

[15] Bolger A.M., Lohse M., Usadel B. (2014). Trimmomatic: A flexible trimmer for Illumina sequence data. Bioinformatics.

[16] Kim D., Paggi J.M., Park C., Bennett C., Salzberg S.L. (2019). Graph-based genome alignment and genotyping with HISAT2 and HISAT-genotype. Nat. Biotechnol..

[17] Anders S., Pyl P.T., Huber W. (2015). HTSeq-A Python framework to work with high-throughput sequencing data. Bioinformatics.

[18] BAQCOM Bioinformatics Analysis for Quality Control and Mapping. https://github.com/hanielcedraz/BAQCOM.

[19] Ritchie M.E., Phipson B., Wu D., Hu Y., Law C.W., Shi W., Smyth G.K. (2015). Limma powers differential expression analyses for RNA-sequencing and microarray studies. Nucleic Acids Res..

[20] R Core Team (2018). A Language and Environment for Statistical Computing.

[21] Benjamini Y., Hochberg Y. (1995). Controllin the false discovery rate: A practical and powerful approch to multiple testing. J. R. Stat. Soc..

[22] Huang D.W., Sherman B.T., Lempicki R.A. (2009). Systematic and integrative analysis of large gene lists using DAVID bioinformatics resources. Nat. Protoc..

[23] Supek F., Bošnjak M., Škunca N., Šmuc T. (2011). Revigo summarizes and visualizes long lists of gene ontology terms. PLoS ONE.

[24] Kaimal V., Bardes E.E., Tabar S.C., Jegga A.G., Aronow B.J. (2010). ToppCluster: A multiple gene list feature analyzer for comparative enrichment clustering and networkbased dissection of biological systems. Nucleic Acids Res..

[25] Shannon P., Markiel A., Ozier O., Baliga N.S., Wang J.T., Ramage D., Amin N., Schwikowski B., Ideker T. (2003). Cytoscape: A Software Environment for Integrated Models. Genome Res..

[26] Dong J., Hu Y., Fan X., Wu X., Mao Y., Hu B., Guo H., Wen L., Tang F. (2018). Single-cell RNA-seq analysis unveils a prevalent epithelial/mesenchymal hybrid state during mouse organogenesis. Genome Biol..

[27] Yu F.X., Guan K.L. (2013). The Hippo pathway: Regulators and regulations. Genes Dev..

[28] Halder G., Johnson R.L. (2011). Hippo signaling: Growth control and beyond. Development.

[29] Zheng Y., Pan D. (2019). The Hippo Signaling Pathway in Development and Disease. Dev. Cell.

[30] Pan D. (2010). The hippo signaling pathway in development and cancer. Dev. Cell.

[31] Fu V., Plouffe S.W., Guan K. (2017). The Hippo pathway in organ development, homeostasis, and regeneration. Curr. Opin. Cell Biol..

[32] Lorthongpanich C., Issaragrisil S. (2015). Emerging Role of the Hippo Signaling Pathway in Position Sensing and Lineage Specification in Mammalian Preimplantation Embryos. Biol. Reprod..

[33] Sharma J., Madan P. (2019). Characterisation of the Hippo signalling pathway during bovine preimplantation embryo development. Reprod. Fertil. Dev..

[34] Karaulanov E., Knöchel W., Niehrs C. (2004). Transcriptional regulation of BMP4 synexpression in transgenic Xenopus. EMBO J..

[35] Tramullas M., Lantero A., Díaz Á., Morchón N., Merino D., Villar A., Buscher D., Merino R., Hurlé J.M., Izpisúa-Belmonte J.C. (2010). BAMBI (bone morphogenetic protein and activin membrane-bound inhibitor) reveals the involvement of the transforming growth factor-β family in pain modulation. J. Neurosci..

[36] Dituri F., Cossu C., Mancarella S., Giannelli G. (2019). The Interactivity between TGFβ and BMP Signaling in Organogenesis, Fibrosis, and Cancer. Cells.

[37] Grotewold L., Plum M., Dildrop R., Peters T., Rüther U. (2001). Bambi is coexpressed with Bmp-4 during mouse embryogenesis. Mech. Dev..

[38] Higashihori N., Song Y., Richman J.M. (2008). Expression and regulation of the decoy bone morphogenetic protein receptor BAMBI in the developing avian face. Dev. Dyn..

[39] Wu M.Y., Hill C.S. (2009). TGF-β Superfamily Signaling in Embryonic Development and Homeostasis. Dev. Cell.

[40] Dagnino L., Fry C.J., Bartley S.M., Farnham P., Gallie B.L., Phillips R.A. (1997). Expression patterns of the E2F family of transcription factors during murine epithelial development. Cell Growth Differ..

[41] Sears R.C., Nevins J.R. (2002). Signaling networks that link cell proliferation and cell fate. J. Biol. Chem..

[42] White J., Stead E., Faast R., Conn S., Cartwright P., Dalton S. (2004). Developmental Activation of the Rb–E2F Pathway and Establishment of Cell Cycle-regulated Cyclin-dependent Kinase Activity during Embryonic Stem Cell Differentiation. Mol. Biol. Cell.

[43] Nagy Z., Tora L. (2007). Distinct GCN5/PCAF-containing complexes function as co-activators and are involved in transcription factor and global histone acetylation. Oncogene.

[44] Indovina P., Pentimalli F., Casini N., Vocca I., Giordano A. (2015). RB1 dual role in proliferation and apoptosis: Cell fate control and implications for cancer therapy. Oncotarget.

[45] Sen R., Pezoa S.A., Shull L.C., Hernandez-Lagunas L., Niswander L.A., Artinger K.B. (2018). Kat2a and Kat2b acetyltransferase activity regulates craniofacial cartilage and bone differentiation in Zebrafish and mice. J. Dev. Biol..

[46] Epstein J.A., Aghajanian H., Singh M.K. (2015). Semaphorin signaling in cardiovascular development. Cell Metab..

[47] Dabbagh A., Amini A., Mohammad-Amin Abdollahifar M.A.S., Dabbagh A., Conte A.H., Lubin L. (2017). Congenital Heart Disease in Pediatric and Adult Patients: Anesthetic and Perioperative Management. Congenital Heart Disease in Pediatric and Adult Patients.

[48] Hu W., Xin Y., Hu J., Sun Y., Zhao Y. (2019). Inhibitor of DNA binding in heart development and cardiovascular diseases. Cell Commun. Signal..

[49] Franciolli A.L.R., Cordeiro B.M., da Fonseca E.T., Rodrigues M.N., Sarmento C.A.P., Ambrosio C.E., de Carvalho A.F., Miglino M.A., Silva L.A. (2011). Characteristics of the equine embryo and fetus from days 15 to 107 of pregnancy. Theriogenology.

[50] McFadden D.G., Barbosa A.C., Richardson J.A., Schneider M.D., Srivastava D., Olson E.N. (2005). The Hand1 and Hand2 transcription factors regulate expansion of the embryonic cardiac ventricles in a gene dosage-dependent manner. Development.

[51] Valenti O., Prima F.A.F.D., Renda E., Faraci M., Hyseni E., Domenico R.D., Monte S., Giorgio E. (2011). Fetal cardiac function during the first trimester of pregnancy. J. Prenat. Med..

[52] Tan C.M.J., Lewandowski A.J. (2019). The Transitional Heart: From Early Embryonic and Fetal Development to Neonatal Life. Fetal Diagn. Ther..

[53] Leiva M.C., Tolosa J.E., Binotto C.N., Weiner S., Huppert L., Denis A.L., Huhta J.C. (1999). Fetal cardiac development and hemodynamics in the first trimester. Ultrasound Obstet. Gynecol..

[54] Teekakirikul P., Kelly M.A., Rehm H.L., Lakdawala N.K., Funke B.H. (2013). Inherited cardiomyopathies: Molecular genetics and clinical genetic testing in the postgenomic era. J. Mol. Diagnostics.

[55] Meadows S.M., Fletcher P.J., Moran C., Xu K., Neufeld G., Chauvet S., Mann F., Krieg P., Cleaver O. (2012). Integration of Repulsive Guidance Cues Generates Avascular Zones that Shape Mammalian Blood Vessels. Circ. Res..

[56] Murakami M. (2012). Signaling required for blood vessel maintenance: Molecular basis and pathological manifestations. Int. J. Vasc. Med..

[57] Kolte D., McClung J.A., Aronow W.S., Aronow W.S., McClung J.A. (2016). Vasculogenesis and Angiogenesis. Translational Research in Coronary Artery Disease: Pathophysiology to Treatment.

[58] Dzierzak E., Philipsen S. (2013). Erythropoiesis: Development and differentiation. Cold Spring Harb. Perspect. Med..

[59] McGrath K.E., Frame J.M., Fromm G.J., Koniski A.D., Kingsley P.D., Little J., Bulger M., Palis J. (2011). A transient definitive erythroid lineage with unique regulation of the β-globin locus in the mammalian embryo. Blood.

[60] Nandakumar S.K., Ulirsch J.C., Sankaran V.G. (2016). Advances in Understanding Erythropoiesis: Evolving Perspectives. Br. J. Haematol..

[61] Lee R., Kertesz N., Joseph S.B., Jegalian A., Wu H. (2001). Erythropoietin (Epo) and EpoR expression and 2 waves of erythropoiesis. Blood.

[62] Sankaran V.G., Xu J., Orkin S.H. (2010). Advances in the understanding of haemoglobin switching. Br. J. Haematol..

[63] Seely J.C. (2017). A brief review of kidney development, maturation, developmental abnormalities, and drug toxicity: Juvenile animal relevancy. J. Toxicol. Pathol..

[64] Moritz K.M., Wintour E.M. (1999). Functional development of the meso- and metanephros. Pediatr. Nephrol..

[65] Georgas K.M., Chiu H.S., Lesieur E., Rumballe B.A., Little M.H. (2011). Expression of metanephric nephron-patterning genes in differentiating mesonephric tubules. Dev. Dyn..

[66] Little M.H., McMahon A.P. (2012). Mammalian kidney development: Principles, progress, and projections. Cold Spring Harb. Perspect. Biol..

[67] Schedl A. (2007). Renal abnormalities and their developmental origin. Nat. Rev. Genet..

[68] Raciti D., Reggiani L., Geffers L., Jiang Q., Bacchion F., Subrizi A.E., Clements D., Tindal C., Davidson D.R., Kaissling B. (2008). Organization of the pronephric kidney revealed by large-scale gene expression mapping. Genome Biol..

[69] Roselli S., Gribouval O., Boute N., Sich M., Benessy F., Attié T., Gubler M.C., Antignac C. (2002). Podocin localizes in the kidney to the slit diaphragm area. Am. J. Pathol..

[70] Wang P., Chen Y., Yong J., Cui Y., Wang R., Wen L., Qiao J., Tang F. (2018). Dissecting the Global Dynamic Molecular Profiles of Human Fetal Kidney Development by Single-Cell RNA Sequencing. Cell Rep..

[71] Karalova E., Semerjyan Z., Manukyan A., Flora I., Panyan N., Karalyan Z., Tatoyan M. (2018). The embryonic development of the pig excretory system. Porcine Res..

[72] Kuure S., Vuolteenaho R., Vainio S. (2000). Kidney morphogenesis: Cellular and molecular regulation. Mech. Dev..

[73] Rehfeldt C., Fiedler I., Dietl G., Ender K. (2000). Myogenesis and postnatal skeletal muscle cell growth as influenced by selection. Livest. Prod. Sci..

[74] Maltin C.A., Delday M.I., Sinclair K.D., Steven J., Sneddon A.A. (2001). Impact of manipulations of myogenesis in utero on the performance of adult skeletal muscle. Reproduction.

[75] Chang K.C. (2007). Key signalling factors and pathways in the molecular determination of skeletal muscle phenotype. Animal.

[76] Chal J., Pourquié O. (2017). Making muscle: Skeletal myogenesis in vivo and in vitro. Development.

[77] Wigmore P.M., Stickland N.C. (1983). Muscle development in large and small pig fetuses. J. Anat..

[78] Tasleem Jan A., Ju Lee E., Ahmad S., Choi I. (2016). Meeting the meat: Delineating the molecular machinery of muscle development. J. Anim. Sci. Technol..

[79] Millay D.P., O’Rourke J.R., Sutherland L.B., Bezprozvannaya S., Shelton J.M., Bassel-Duby R., Olson E.N. (2013). Myomaker: Amembrane activator of myoblast fusion and muscle formation. Nature.

[80] Wang J., Feng C., Liu T., Shi M., Wu G., Bazer F.W. (2017). Physiological alterations associated with intrauterine growth restriction in fetal pigs: Causes and insights for nutritional optimization. Mol. Reprod. Dev..

[81] Allard J.B., Duan C. (2018). IGF-binding proteins: Why do they exist and why are there so many?. Front. Endocrinol. (Lausanne)..

[82] DiGirolamo D.J., Kiel D.P., Esser K.A. (2013). Bone and Skeletal Muscle: Neighbors With Close Ties. J. Bone Miner. Res..

[83] Olsen B.R., Reginato A.M., Wang W. (2000). BONE DEVELOPMENT. Annu. Rev. Cell Dev. Biol..

[84] Long F., Ornitz D.M. (2013). Development of the endochondral skeleton. Cold Spring Harb. Perspect. Biol..

[85] Kato M., Patel M.S., Levasseur R., Lobov I., Chang B.H.J., Glass D.A., Hartmann C., Li L., Hwang T.H., Brayton C.F. (2002). Cbfa1-independent decrease in osteoblast proliferation, osteopenia, and persistent embryonic eye vascularization in mice deficient in Lrp5, a Wnt coreceptor. J. Cell Biol..

[86] Day T.F., Guo X., Garrett-Beal L., Yang Y. (2005). Wnt/β-catenin signaling in mesenchymal progenitors controls osteoblast and chondrocyte differentiation during vertebrate skeletogenesis. Dev. Cell.

[87] Rutkovskiy A., Stensløkken K.-O., Vaage I.J. (2016). Osteoblast Differentiation at a Glance. Med. Sci. Monit. Basic Res..

[88] McKee M.D., Cole W.G. (2012). Bone Matrix and Mineralization. Pediatric Bone.

[89] Qin C., Brunn J.C., Cook R.G., Orkiszewski R.S., Malone J.P., Veis A., Butler W.T. (2003). Evidence for the proteolytic processing of dentin matrix protein 1. J. Biol. Chem..

[90] Ninomiya K., Miyamoto T., Imai J.I., Fujita N., Suzuki T., Iwasaki R., Yagi M., Watanabe S., Toyama Y., Suda T. (2007). Osteoclastic activity induces osteomodulin expression in osteoblasts. Biochem. Biophys. Res. Commun..

[91] Qin C., D’Souza R., Feng J.Q. (2007). Dentin Matrix Protein 1 (DMP1): New and important roles for biomineralization and phosphate homeostasis. J. Dent. Res..

[92] Van Der Lende T., Van Rens B.T.T.M. (2003). Critical periods for foetal mortality in gilts identified by analysing the length distribution of mummified foetuses and frequency of non-fresh stillborn piglets. Anim. Reprod. Sci..

[93] Gelse K., Pöschl E., Aigner T. (2003). Collagens - structure, function, and biosynthesis. Adv. Drug Deliv. Rev..

[94] Jabłońska-Trypuć A., Matejczyk M., Rosochacki S. (2016). Matrix metalloproteinases (MMPs), the main extracellular matrix (ECM) enzymes in collagen degradation, as a target for anticancer drugs. J. Enzyme Inhib. Med. Chem..

[95] Sabatelli P., Gualandi F., Gara S.K., Grumati P., Zamparelli A., Martoni E., Pellegrini C., Merlini L., Ferlini A., Bonaldo P. (2012). Expression of collagen VI α5 and α6 chains in human muscle and in Duchenne muscular dystrophy-related muscle fibrosis. Matrix Biol..

[96] Lefebvre V., Smits P. (2005). Transcriptional control of chondrocyte fate and differentiation. Birth Defects Res. Part C Embryo Today Rev..

[97] Ortega N., Behonick D.J., Werb Z. (2004). Matrix remodeling during endochondral ossification. Trends Cell Biol..

[98] Mackie E.J., Ahmed Y.A., Tatarczuch L., Chen K.S., Mirams M. (2008). Endochondral ossification: How cartilage is converted into bone in the developing skeleton. Int. J. Biochem. Cell Biol..

[99] Liang H.P.H., Xu J., Xue M., Jackson C. (2016). Matrix metalloproteinases in bone development and pathology: Current knowledge and potential clinical utility. Met. Med..

[100] Jin X. (2016). The role of neurogenesis during development and in the adult brain. Eur. J. Neurosci..

[101] Martynoga B., Drechsel D., Guillemot F. (2012). Molecular control of neurogenesis: A view from the mammalian cerebral cortex. Cold Spring Harb. Perspect. Biol..

[102] Lee J.E. (1997). Basic helix-loop-helix genes in neural development. Curr. Opin. Neurobiol..

[103] Kim E.J., Hori K., Wyckoff A., Dickel L.K., Koundakjian E.J., Goodrich L.V., Johnson J.E. (2011). Spatiotemporal Fate Map of Neurogenin1 (Neurog1) Lineages in the Mouse Central Nervous System. J. Comp. Neurol..

[104] Hwang H.M., Ku R.Y., Hashimoto-Torii K. (2019). Prenatal Environment That Affects Neuronal Migration. Front. Cell Dev. Biol..

[105] Luján R., Shigemoto R., López-Bendito G. (2005). Glutamate and GABA receptor signalling in the developing brain. Neuroscience.

[106] Cuzon V.C., Yeh P.W., Cheng Q., Yeh H.H. (2006). Ambient GABA promotes cortical entry of tangentially migrating cells derived from the medial ganglionic eminence. Cereb. Cortex.

[107] Manent J., Represa A. (2007). Neurotransmitters and Brain Maturation: Early Paracrine Actions of GABA and Glutamate Modulate Neuronal Migration. Neuroscientist.

[108] Barber M., Di Meglio T., Andrews W.D., Hernández-Miranda L.R., Murakami F., Chédotal A., Parnavelas J.G. (2009). The role of Robo3 in the development of cortical interneurons. Cereb. Cortex.

[109] Silbereis J.C., Pochareddy S., Zhu Y., Li M., Sestan N. (2016). The Cellular and Molecular Landscapes of the Developing Human Central Nervous System. Neuron.

[110] Farhy-Tselnicker I., Allen N.J. (2018). Astrocytes, neurons, synapses: A tripartite view on cortical circuit development. Neural Dev..

[111] Borsani E., Della Vedova A.M., Rezzani R., Rodella L.F., Cristini C. (2018). Correlation between human nervous system development and acquisition of fetal skills: An overview. Brain Dev..

[112] Chen V.S., Morrison J.P., Southwell M.F., Foley J.F., Bolon B., Elmore S.A. (2017). Histology Atlas of the Developing Prenatal and Postnatal Mouse Central Nervous System, with Emphasis on Prenatal Days E7.5 to E18.5. Toxicol. Pathol..

