# Supplementary material for: Transcription Landscape of the Early Developmental Biology in Pigs
Source: Animals (Basel). 2021 May 18;11(5):1443. doi: 10.3390/ani11051443 (PMC8157595; doi:10.3390/ani11051443)
Supplement: Supplementary file 1 [file animals-11-01443-s001.zip › animals-1197879-supplementary/animals-1197879- S1 - M&M- conversion.pdf]

animals

MDPI

1. Materials and Methods—Additional Information

1.1. *Animal Experimental design*

The experimental protocol has followed ethical principles in animal research (CONCEA, 2016) and was approved by the Ethical Committee on Animal Use of Universidade Federal de Viçosa (UFV), Minas Gerais, Brazil [protocol no. 06/2017].

A total of 23 commercial line gilts (crossbred females from a Landrace and a Large White pure lines) were inseminated 12 and 24 h after the beginning of fourth estrus. The first insemination day was considered day zero of gestation and the supply of experimental diets occurred 24 h after the second insemination [1,2]. The semen doses were collected from two commercial line boars, with proven reproductive performance (semen analyses), according to the requirements for use in pig artificial insemination (AI) programs recommended by Colégio Brasileiro de Reprodução Animal [3]. For each female, semen from the same boar was used in the two inseminations. A total of 11 gilts were fed a control diet (CON) and 12 gilts were fed the CON diet supplemented with 1.0% L-arginine (ARG), using a completely randomized experimental design with two diets (CON and ARG), and two gestational ages were considered (25 and 35 days of gestation). At the beginning of the experimental period, the mean body weights of 25-days females were 154.00 ± 3.15 kg and 152.10 ± 7.12 kg for CON and ARG, respectively, and for the 35-days females, at the same period, the mean body weights were 143.15 ± 8.96 kg and 148.55 ± 4.71 kg for CON and ARG, respectively. As described in Costa et al. [1] and Garcia et al. [2], the experimental CON diet were mainly composed of corn, soybean meal, mineral and vitamins supplements, formulated to meet the nutritional requirements of females during gestation [4], and the ARG diet was formulated by replacing clay filler by L-arginine.

From the 23 inseminated gilts, 20 became pregnant. A total of five CON and five ARG gilts (at 25 days of gestation) and four CON and six ARG gilts (at 35 days of gestation) were rendered unconscious using head-only electrical stunning (240V, 1.3A) and immediately exsanguinated. After slaughter, four conceptuses were collected per female at each gestational age. The conceptuses were quickly washed with PBS (Phosphate Buffered Saline) solution, individually identified, stored in liquid nitrogen, and transported to the Animal Biotechnology Laboratory (LABTEC) at the Department of Animal Science, UFV. At LABTEC, each conceptus was entirely and separately macerated in liquid nitrogen. From these, three embryos from 3 CON gilts and three embryos from 3 ARG gilts at 25 days of gestation and three fetuses from 3 CON gilts and three fetuses from 3 ARG gilts at 35 days of gestation were randomly chosen for RNA-seq analysis, totalizing 36 samples, and transported in liquid nitrogen to the Animal Genetics Laboratory at the Embrapa Swine and Poultry Nacional Research Center, Concordia, SC, Brazil, for further RNA extraction and library preparation.

Since in the current study we aimed to investigate the transcriptional changes that occur during the development stages instead of the effects of L-arginine supplementation, only data from embryos and fetuses from non-supplemented gilts (n = 3 embryos from each three non-supplemented gilts at 25 days of gestation and n = 3 fetuses from each three non-supplemented gilts at 35 days of gestation) were used for the analyses. At the beginning of the experimental period, the mean body weights of non-supplemented commercial females from the 25 and 35 days of gestation groups were 156.27 ± 3.05 Kg and 136.93 ± 6.25 Kg, respectively.

Tables S1–S5. are provided separately, attached as an Excel File.

References

1. Costa, K.A.; Saraiva, A.; Guimarães, J.D.; Marques, D.B.D.; Machado-Neves, M.; Barbosa, L.M.R.; Villadiego, F.A.C.; Veroneze, R.; De Oliveira, L.F.; Garcia, I.S.; et al. Dietary L-arginine supplementation during early gestation of gilts affects conceptuses development. *Theriogenology* **2019**, *140*, 62–71, doi:10.1016/j.theriogenology.2019.08.018.
2. Garcia, I.S.; Teixeira, S.A.; Costa, K.A.; Marques, D.B.D.; Rodrigues, G.D.A.; Costa, T.C.; Guimarães, J.D.; Otto, P.I.; Saraiva, A.; Ibelli, A.M.G.; et al. l-Arginine supplementation of gilts during early gestation modulates energy sensitive pathways in pig conceptuses. *Mol. Reprod. Dev.* **2020**, 1–16, doi:10.1002/mrd.23397.
3. Colégio Brasileiro De Reprodução Animal. *Manual para exame andrológico e avaliação de sêmen animal*; 3rd ed.; Minas Gerais: Belo Horizonte, MG, Brazil, 2013.
4. Rostagno, H.S.; Albino, L.F.T.; Donzele, J.L.; Gomes, P.C.; De Oliveira, R.F.; Lopes, D.C.; Ferreira, A.S.; Barreto, S.L. de T.; Euclides, R.F. *Brazilian tables for poultry and swine: Composition of feedstuffs and nutritional requirements;* 3rd ed.; Universidad Federal de Viçosa-Departamento de Zootecnia: Viçosa, MG, Brazil, 2011, p. 251.
